# Supporting scale-up of COVID-19 RT-PCR testing processes with discrete event simulation

**DOI:** 10.1371/journal.pone.0255214

**Published:** 2021-07-29

**Authors:** Jad El Hage, Patti Gravitt, Jacques Ravel, Nadia Lahrichi, Erica Gralla

**Affiliations:** 1 Department of Engineering Management and Systems Engineering, George Washington University, Washington, DC, United States of America; 2 Department of Epidemiology and Public Health, University of Maryland School of Medicine, Baltimore, Maryland, United States of America; 3 Institute for Genome Sciences and Department of Microbiology and Immunology, University of Maryland School of Medicine, Baltimore, Maryland, United States of America; 4 Department of Mathematics and Industrial Engineering, CIRRELT & Polytechnique Montreal, Montreal, Québec, Canada; Waseda University: Waseda Daigaku, JAPAN

## Abstract

Testing is critical to mitigating the COVID-19 pandemic, but testing capacity has fallen short of the need in the United States and elsewhere, and long wait times have impeded rapid isolation of cases. Operational challenges such as supply problems and personnel shortages have led to these bottlenecks and inhibited the scale-up of testing to needed levels. This paper uses operational simulations to facilitate rapid scale-up of testing capacity during this public health emergency. Specifically, discrete event simulation models were developed to represent the RT-PCR testing process in a large University of Maryland testing center, which retrofitted high-throughput molecular testing capacity to meet pandemic demands in a partnership with the State of Maryland. The simulation models support analyses that identify process steps which create bottlenecks, and evaluate “what-if” scenarios for process changes that could expand testing capacity. This enables virtual experimentation to understand the trade-offs associated with different interventions that increase testing capacity, allowing the identification of solutions that have high leverage at a feasible and acceptable cost. For example, using a virucidal collection medium which enables safe discarding of swabs at the point of collection removed a time-consuming “deswabbing” step (a primary bottleneck in this laboratory) and nearly doubled the testing capacity. The models are also used to estimate the impact of demand variability on laboratory performance and the minimum equipment and personnel required to meet various target capacities, assisting in scale-up for any laboratories following the same process steps. In sum, the results demonstrate that by using simulation modeling of the operations of SARS-CoV-2 RT-PCR testing, preparedness planners are able to identify high-leverage process changes to increase testing capacity.

## Introduction

The COVID-19 pandemic is rapidly evolving, having already claimed more than half a million deaths in the United States alone and more than 2.5 million worldwide [1]. Testing is an effective tool in containment of the pandemic, allowing cases to be identified and isolated before they can spread the disease further. Even as vaccination is rolled out, testing will remain essential: vaccines are not yet approved for children yet schools must reopen, many parts of the world remain unvaccinated, and worrisome variants threaten future surges [2].

Unfortunately, testing capacity has fallen far short of the need for containing the pandemic in the United States. An estimated 300 million COVID tests per month are needed for reopening K12 schools alone [3], yet only about 39 million are being conducted per month, down from a peak of 60 million in mid-January 2021 [2]. Moreover, there have been long wait times for test results, sometimes more than a week, which undermines the value of testing -- the ability to rapidly identify and isolate active cases [4].

The shortage of testing in the United States was due at least partially to operational challenges with the laboratory aspects of PCR testing (the gold standard for sensitive detection of symptomatic and asymptomatic cases), including supply problems with essential components like reagents and instruments, shortages of qualified personnel, and the sheer complexity of organizing the process [3, 5–7]. To meet these challenges, multiple strategies have been explored, including the prioritization of scarce tests for particular populations [8–10], alternative approaches for nucleic acid testing [5], and pooling multiple samples together for testing [e.g., 11, 12]. However, other than pooling, innovations in the *operational processes* of COVID testing have received very little attention. Two studies highlight opportunities for improving processes through drive-through sample collection [13, 14]. They and others [15] argue that process optimization could enable increased efficiency and, thus, scale-up of testing. Despite these clear opportunities, however, we are not aware of any investigations of the laboratory processes for PCR testing.

This paper examines the laboratory operational processes associated with PCR testing, through the development and validation of a discrete event simulation model to represent SARS-CoV-2 RT-PCR testing processes at the University of Maryland Pathology Associates/Maryland Genomics (UMPA/MG), a major academic testing center for the state of Maryland which retrofitted high-throughput molecular testing capacity to meet pandemic demands in a partnership with the State of Maryland. The model represents the cumulative impact of multiple interacting process steps. “What if” scenarios were devised to represent various potential process and resource configurations, then evaluated to predict their relative performance on several key performance indicators, including the result turnaround time and the total number of samples that can be processed per week. With this approach, we investigate (1) how to eliminate successive bottlenecks in the UMPA/MG laboratory to increase its capacity; (2) how variability in demand affects laboratory performance; and (3) equipment, personnel, and process configurations to meet several target levels of laboratory capacity. The results enabled decision-makers to test system changes in silico before making major investments or reorganization. This work demonstrates the value of simulation to support process optimization for scaling up testing capacity to meet the needs of the current COVID-19 pandemic and to plan ahead for future public health emergencies.

## Background

SARS-CoV-2 PCR testing is now performed by a wide variety of commercial, academic, and public organizations in the United States, many of whom significantly ramped up their operations to respond to the public health needs. This paper studies one of these organizations, and later considers how the resulting insights may be generalized to other organizations and support pandemic preparedness planning.

### Testing process and context

In late February 2020, the University of Maryland recognized an emergent need for rapid expansion of SARS-CoV-2 testing to meet the public health demand for pandemic surveillance and response. Over the next 3 months, the Microbiome Service Laboratory at the Institute for Genome Sciences, in collaboration with the University of Maryland Pathology Associates (UMPA) and the Maryland Department of Health, scaled-up their high-throughput genomic research laboratory operations. UMPA/MG is now a CLIA-certified high-complexity laboratory.

The UMPA/MG adapted and implemented a RT-PCR test to detect SARS-CoV-2 in samples collected from nasopharyngeal or nasal swabs. This test is a high-throughput version of the CDC 2019-nCoV Realtime RT-PCR test. It is authorized under the Emergency Use Authorization (EUA) by the FDA and has performance characteristics that were established in accordance with CLIA regulations. The test uses an assay that comprises three separate reactions that measure the amount of two parts of the virus, the N1 and N3 regions of the N gene, and of a human gene, RNaseP, which is used to assure the quality and integrity of the samples collected. The test has been validated for samples collected in a variety of transport buffers as well as self-collection.

**Fig 1:**
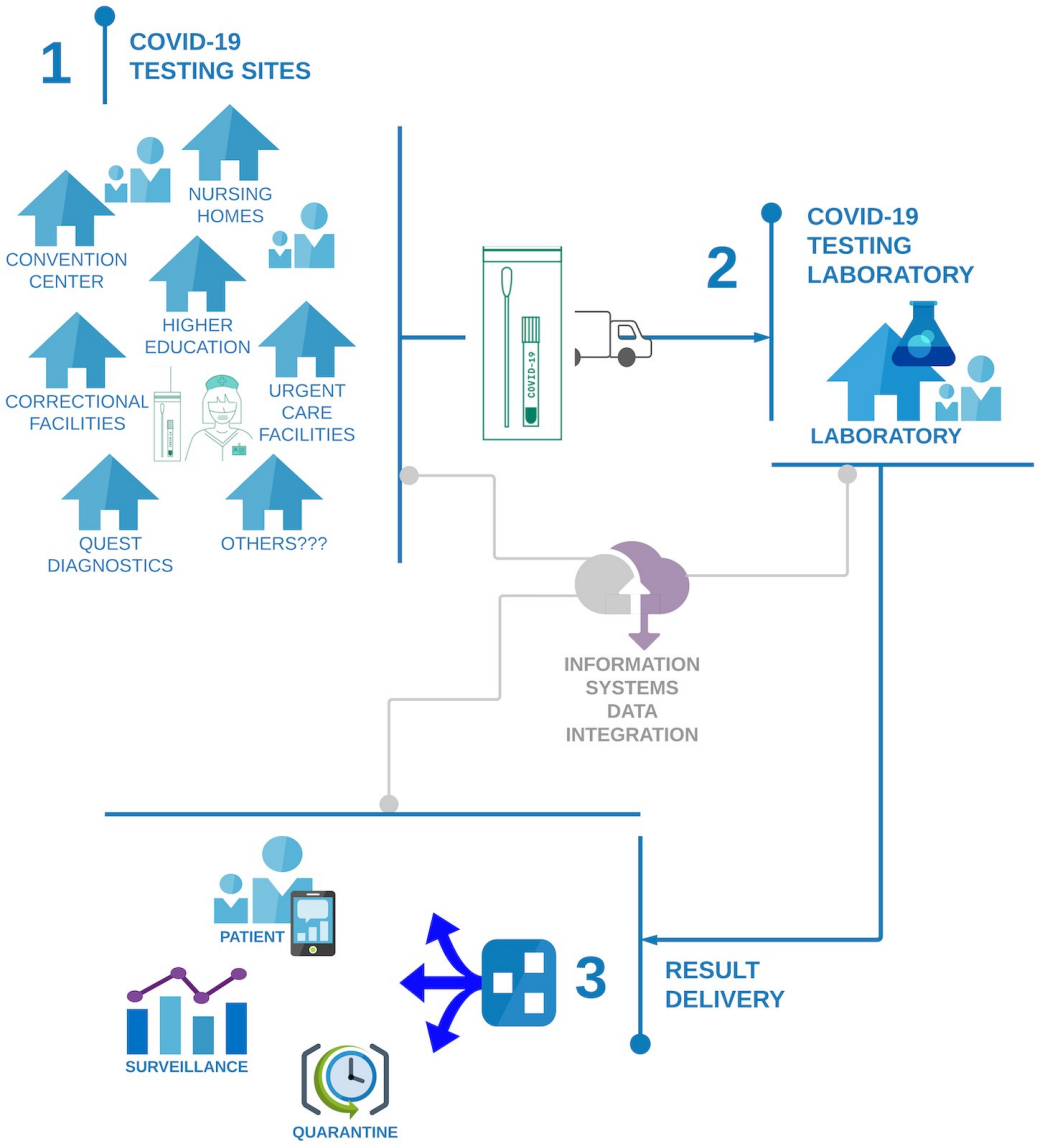
The testing ecosystem. Schematic representation of the SARS-CoV-2 testing system, including 1) a variety of community and facility sites collecting samples for testing, which are transported to 2) a centralized SARS-CoV-2 testing laboratory, which completes the test and 3) delivers results back to the individual through multiple platforms. Information systems link the different sectors.

SARS-CoV-2 RT-PCR testing is a complex ecosystem involving many stakeholders and many different interrelated processes, illustrated in Fig 1. UMPA/MG provides testing for a broad spectrum of clients, including nursing homes, correctional facilities, University of Maryland campuses and other private colleges and universities, urgent care practices, ‘spill-over’ from commercial laboratories, and public health testing sites. Samples are collected in each of these settings, then transported to the laboratory for testing. The results are delivered to clients and used for many purposes, including patient care, public health surveillance, and choices about quarantine. All of these processes are supported by different information systems that share data across these sites.

**Fig 2:**
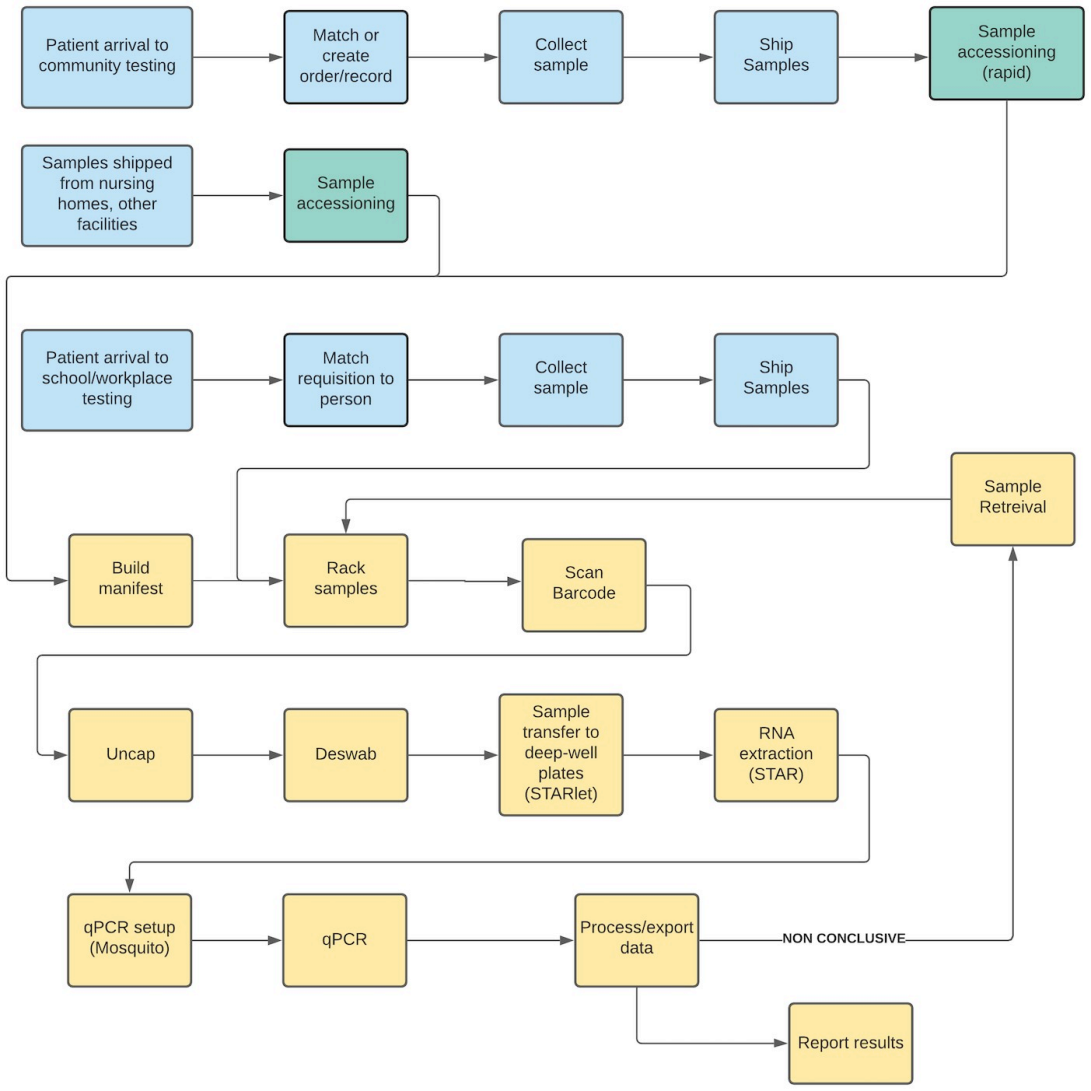
Sample collection and testing process. Steps performed at sample collection sites are shown in blue, sample accessioning in green, and laboratory steps in yellow.

This paper focuses on the middle portion of this ecosystem: the laboratory itself and its interfaces with the other elements of the system. Fig 2 shows the detailed sample collection and testing process. Samples are first collected and shipped to the laboratory (blue boxes in Fig 2). The specifics of this process differ across all the different collection sites. The figure details the three primary processes. (1) At public community testing centers, such as the Baltimore Convention Center, electronic orders are created in the medical information system. (2) At universities, records are created ahead of time and simply matched to the testee when he or she arrives for testing. (3) At nursing homes, correctional facilities, and other independent facilities, different types of paper or electronic records may be used. In all cases, samples are collected with nasal or nasopharyngeal swabs; the swabs are inserted into sample tubes, and the tubes are capped and transported to the laboratory.

Next, accessioning (green boxes in Fig 2) involves matching the sample to a record in the medical information system, and/or creating a record if it does not yet exist. This can be a very time-consuming process, depending on the availability and validity of the paper or electronic record for the sample. Accessioning is straightforward for samples from universities, whose records were already created in the laboratory’s information system, and for community testing, where electronic orders can be imported from the medical record system. However, it can be very labor-intensive for other independent facilities, where paper records may be illegible or contain mistakes, and electronic records may need to be created at the laboratory.

**Fig 3:**
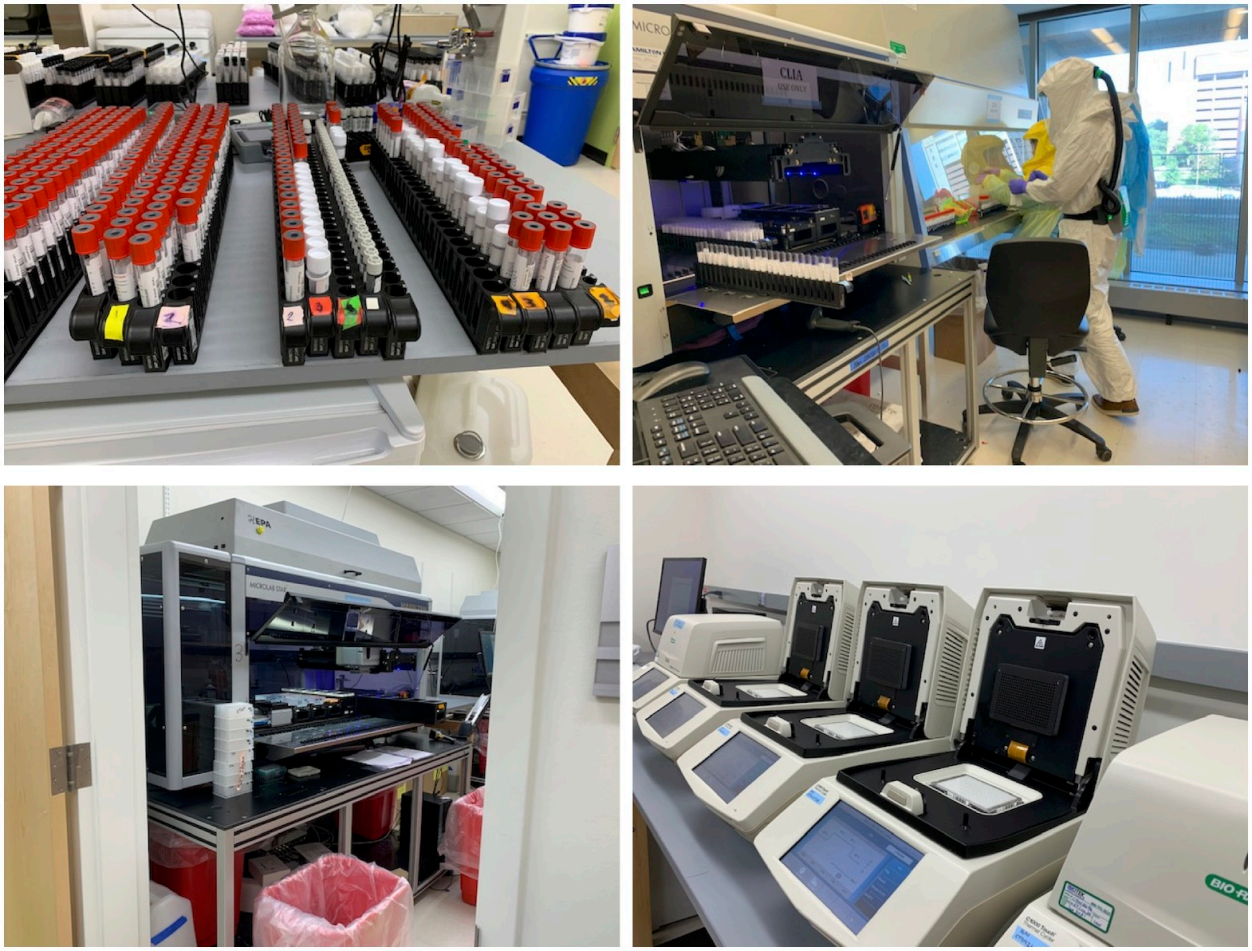
Laboratory process steps. (Top left) Samples racked and scanned; note the many different sizes and shapes. (Top right) Tubes are uncapped and deswabbed manually then placed on the deck of the Hamilton STARlet for transfer to deep-well plates. (Bottom left) Deep-well plates stacked in front of a Hamilton STAR for RNA extraction. (Bottom right) qPCR in progress and completed.

After accessioning, the laboratory testing process begins. These steps are shown in yellow in Fig 2, and Fig 3 provides photos of some of them. Samples are put into long racks and the barcodes on each sample tube are scanned into the laboratory information management system (LIMS). Next, the sample tubes are uncapped and the swabs are removed from the tubes (i.e., the samples are “deswabbed”). This is a manual process that is tedious, and can be difficult and time-consuming depending on the size of the swabs and tubes as well as the experience of the technician. Fig 3 illustrates the variability in the shapes and sizes of tubes that might be sent to the laboratory. While uniform requirements for the sample collection would be preferred, supply chain shortages have made it difficult to source sufficient quantities of the most preferred equipment and supplies and thus a requirement to accept a variety of sample types. The racks of tubes are then loaded onto a Hamilton STARlet automated liquid handling robot which will transfer a 200μl aliquot of the sample to a single well in a 96-well plate. Filled deep-well plates are transferred (manually) to a Hamilton STAR automated liquid handling robot (a larger instrument than the STARlet) for RNA extraction. In this process, RNA are eluted into a 96-well plate in the same well location as the originating plate. After RNA extraction, these new plates are processed through a Mosquito automated liquid handling robot with the ability to handle sub-microliter scale volume, to set up the qPCR reactions into 384-well plates. Each RNA sample is divided among three wells used for each of the N1, N3 and RNaseP targets. Using this scheme, a maximum of 96 samples/controls can be processed into one 384-well plate. These 384-well plates are then loaded into a thermocycler instrument where the target RNA is reversed transcribed, amplified and detected. After completion, the data are exported, analyzed and the results reported. Any samples that are inconclusive, i.e., where the human gene is not detected or where there are other problems, trigger the laboratory to retrieve the original sample and retest it.

### Challenges

The UMPA/MG laboratory, like many other academic, government, and commercial laboratories, faced challenges in meeting the demands of the COVID-19 pandemic. Operating procedures had to be adapted for new and diverse clients and the throughput and turnaround time requirements for public health surveillance efforts. Bottlenecks at the interfaces of the system elements (Fig 1) and within the UMPA/MG laboratory were encountered and required rapid troubleshooting.

**Fig 4:**
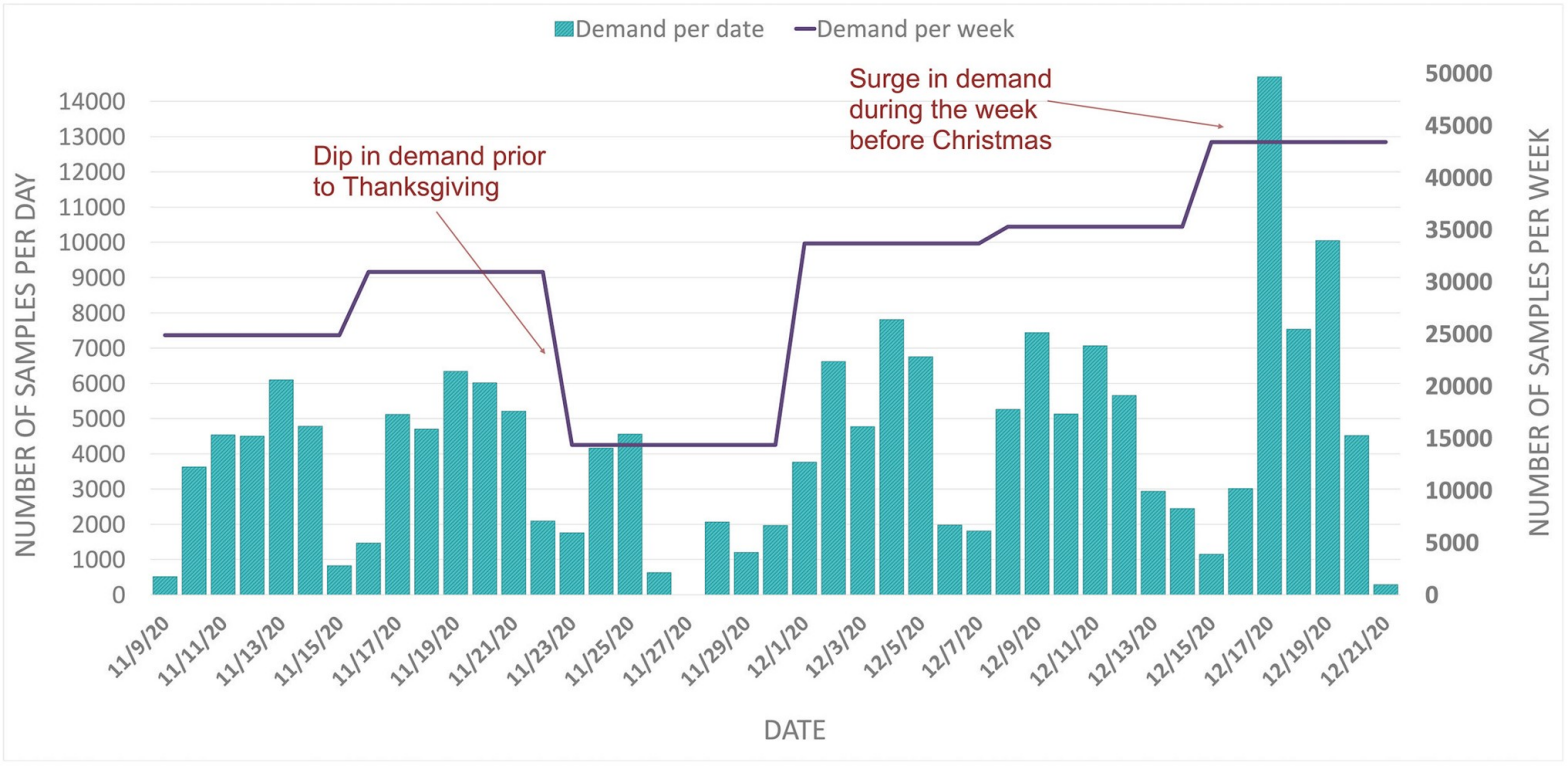
Number of samples arriving for testing at UMPA/MG weekly (line) and daily (bars) during a six-week period in November and December 2020.

A key challenge was dealing with the fluctuations and surges in COVID-19 testing demand and, consequently, managing the implications of this variability on the laboratory operations. Fig 4 shows the total daily and weekly numbers of samples that arrived at the laboratory for testing during a six-week period in November and December 2020. The weekly demand ranged from a low of around 15,000 samples over the Thanksgiving holiday to a high of around 44,000 samples in the week leading up to Christmas, when the region experienced a significant surge in cases. The daily demand also varied between different days of the week, with Fridays typically experiencing the largest-demand followed by low-demand weekends.

**Fig 5:**
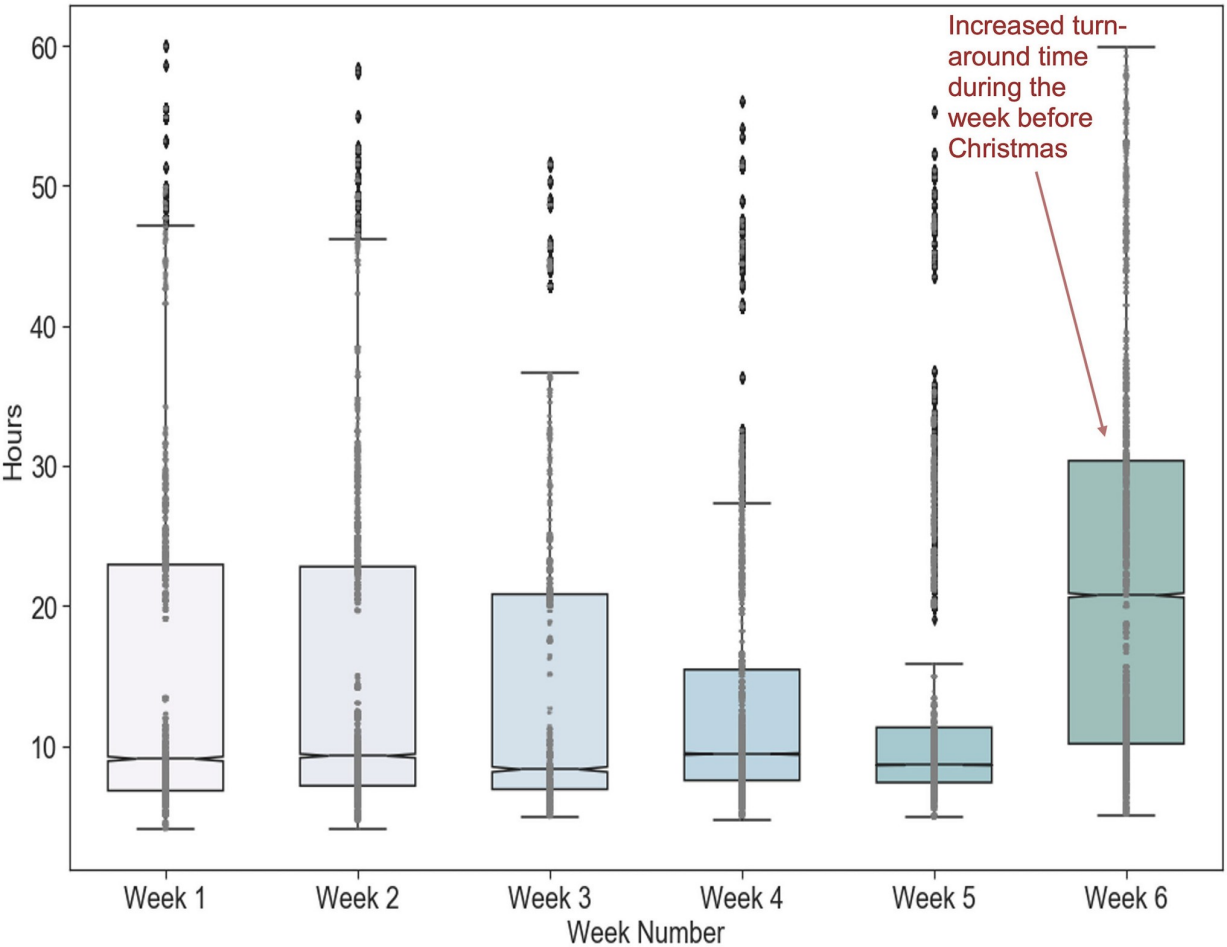
Time to return results at UMPA/MG during a six-week period in November and December 2020.

This erratic sample volume impacted UMPA/MG’s operations because it required rapid adjustments in resources to meet this varying demand. For example, the significant increase in weekly demand during the 2020 holiday season (shown in Fig 4) required sourcing additional essential supplies that had already been difficult to acquire during the pandemic (such as plastic tips and deep-well plates) and required already-stretched staff to work extended shifts. The fluctuation in daily demand meant that it was difficult to predict the number of samples that would be arriving each day, and therefore it was difficult to set staff schedules and plan supplies to meet each day’s demand. These challenges, in turn, affected the time it took to return test results, since days with large demand might create backlogs of samples waiting for processing, while capacity sits idle on low-demand days. Fig 5 shows the variability in turnaround times during the same six weeks leading up to the holidays, and highlights how the large surge in the week before Christmas led to increased and more variable turnaround times. This laboratory was able to keep turnaround times around 24–48 hours in most cases, but many commercial laboratories had much higher turnaround times, suggesting that they suffered from similar but more acute problems.

## Methodology

### Model

A discrete event simulation (DES) model was developed to represent the UMPA/MG testing operations depicted in Fig 2. It simulates the flow of samples through the system, including queues where samples must wait when the next processing step is busy, and variability in both the demand for testing and the time taken by processing steps. As such, it provides an estimate of laboratory capacity and result turnaround time that takes into account the operational difficulties of implementing the testing process. The model contains two major blocks. Block 1 includes the processes at the collection sites (patients arrive for testing and samples are collected, shown in blue in Fig 2), and Block 2 entails the accessioning, laboratory, and result reporting procedures in the laboratory (shown in green and yellow in Fig 2).

Each of these blocks consists of *entities*, *process steps*, *connectors*, *resources* and *batching nodes*. The model represents patients and samples as *entities*. Patients become samples once the samples are collected in the collection sites. These entities flow through the *process steps* (i.e., sample accessioning, deswabbing, etc.) and are transferred from one *process step* to another, following a sequence, via probability-weighted *connectors*. At each process step, machines and technicians process these entities, following a certain work schedule; these are modeled as *resources*. Each of these *resources* has a capacity (e.g., number of machines, capacity of each machine, number of technicians, etc.). Finally, in between some of the process steps, *batching nodes* are used to model the batching of samples that takes place in the laboratory. Since samples may need to wait to be processed, *queues* are defined at each process step. A ranking rule of *first in first out* (FIFO) at each *queue* in a *process step* is assigned, so that samples are processed in the same order that they arrive. Thus, the simulation model represents the flow of samples through the multiple steps included in the testing and lab processing systems (Fig 2).

The simulation model was developed in SIMIO, a commercially available DES package.

### Parameters and key performance indicators

The following types of input parameters are defined: (1) patient and sample arrival rates; (2) processing times for each process step; (3) resource capacities per process step; (4) shift length and work schedules; (5) positivity rate; (6) inconclusive test rate; (7) demand surge factors; (8) batch sizes. Each of these parameters was estimated through the analysis of historical data, on-site observations, and stakeholder estimates. Many of these parameters are represented by distributions rather than fixed values, to represent realistic variability, for example, how often patients arrive for testing, or how long it takes to remove a swab from a sample tube. The values for these parameters are given in Table 1 and/or in S1 File.

**Table 1:** Baseline parameters.

Parameter	Assumption(s)
Sample arrivals	Poisson arrival process, with approximately 26,800 arriving per week
Sample accessioning (nursing homes and similar facilities) processing time and capacity	3–5 minutes/sample; 33 technicians
Sample accessioning (community testing) processing time and capacity	30 seconds/sample; 33 technicians
Uncapping processing time and capacity	1–2 seconds/sample; 2 technicians
Deswabbing processing time and capacity	3–30 seconds/sample (varies with type of swab); 2 technicians
Sample transfer to deep-well plates (STARlet) processing time and capacity	15 minutes per plate of 96 samples; 3 instruments
RNA extraction (STAR) processing time and capacity	1.5 hours/batch; 6 instruments
qPCR processing time, capacity and batch size	1.25 hours/batch; 16 instruments; 96 samples per batch
Lab shift length	8 hours per day

Three main key performance indicators (KPIs) for the testing system were developed. These KPIs were determined based on the most important ways the laboratory measures its performance and the information needed to identify bottlenecks and improve the process. The KPIs are: (1) total number of tests conducted per week; (2) average turn-around time (TAT) to return results from sample collection; and (3) percent utilization of resources (such as personnel or robots) at each step in the process.

The simulation model enables estimates of the performance of the laboratory under different sets of hypothetical conditions (scenarios). For a single scenario, such as the baseline inputs described in Table 1, the model estimates the KPIs by simulating the arrival of thousands of patients at random times during one week of operations, then representing all the process steps that occur for each patient and their sample, including the usage of instruments and technician time, the time elapsed at each step, and any queues that build up while samples are waiting. The model is run multiple times so that a distribution and summary statistics can be computed for the KPIs. Then, the input parameters can be changed to represent a different scenario (for example, a longer work shift) and the process repeated, to determine whether the performance improves for this alternative “what-if” scenario.

For each scenario, 100 runs are performed, and the KPIs are computed from the average across the runs. Specifically, (1) the total number of tests conducted in one week is reported directly by the model. (2) The average TAT is computed by subtracting, for each sample, the time at which the sample was collected from the time at which the result was returned, then averaging across all the samples in each run. (3) The percent utilization of resources is computed by dividing the total amount of time each resource was busy by the total amount of time each resource was available (whether busy or not). For example, each STAR robot is considered available for 8 hours per day (the length of the work shift). It is considered “busy” only when it is processing plates. If there is one STAR robot, and it processes four batches which each requires 1.5 hours, then it is busy for six hours, its availability is 8 hours, and its percent utilization is 75%.

### Verification and validation

Verification and validation ensures that the model represents the process and meets stakeholder needs. First, each step of the model was verified to match the process depicted in Fig 2. Second, the model was validated through (a) comparing the model’s simulated output to empirical data from the laboratory and (b) validating the results with stakeholders.

**Fig 6:**
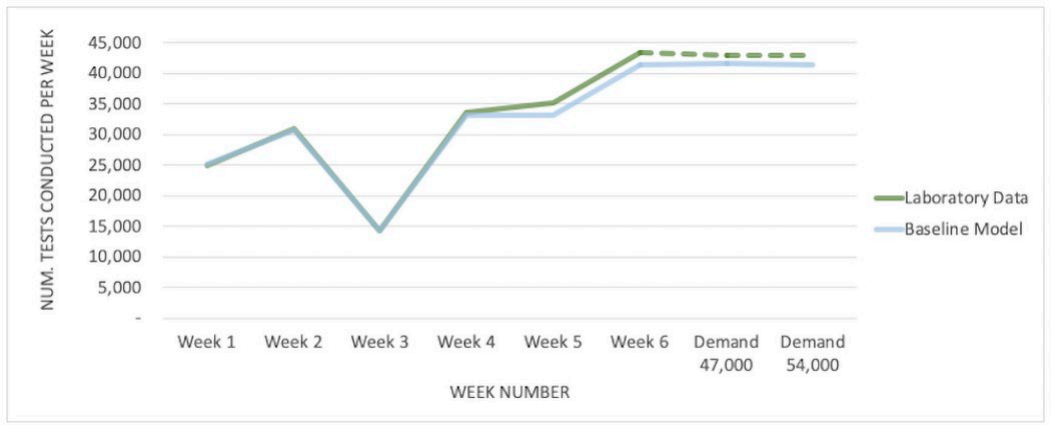
Example of validation results. The model reproduces the empirical data from six weeks of varying demand and two hypothetical higher-demand scenarios.

Regarding the first step (a), two performance indicators were used in this comparison: number of tests conducted per week and result turn-around time. The model closely reproduces the median turnaround time and the total number of tests conducted in 6 weeks of laboratory operations, and it reaches its maximum capacity at roughly the same number of tests per week. Fig 6 is provided as an example of the validation process: it compares the model’s simulated output to the empirical data for the number of tests per week in each of six weeks of real operations and two hypothetical higher-demand scenarios (to validate that the model reproduces the laboratory’s maximum weekly testing capacity).

Regarding the second step (b), we conducted a workshop with stakeholders from across the laboratory. They validated the assumptions on inputs (such as processing times and number of resources) and stated that the model outputs were reasonable, based on their daily experiences in the laboratory. They also confirmed that the first bottleneck identified by the model (see the results section, below) is also seen as a bottleneck by the stakeholders.

## Scenarios and results

To identify opportunities for scaling up testing capacity and improving the process efficiency, we performed several analyses: (1) identifying and eliminating successive bottlenecks in the UMPA/MG laboratory to increase the overall capacity; (2) examining the impact of variability in demand on laboratory performance; and (3) determining the minimum equipment, personnel, and process configurations to meet several target levels of laboratory capacity. Each analysis includes a series of “what-if” scenarios that are evaluated using the DES model under different assumptions about the process and resource configuration and the demand for testing.

The baseline assumptions in each case were the same, except where noted. These baseline assumptions represent the current operations of the UMPA/MG laboratory. Sample arrivals follow a Poisson process which is adjusted to simulate various levels of demand for testing. Other baseline assumptions are listed in Table 1, and the full set of assumptions for the model is provided in the tables in S1 File. To evaluate each “what-if” scenario, the model was run for one week with a warm-up period of 8 hours.

### Analysis 1: Scaling up capacity by eliminating bottlenecks

The first analysis examines the current process at the UMPA/MG laboratory to identify the bottlenecks that constrain capacity and find ways to alleviate them. The results show what process changes or investments would be required to meet a given target weekly testing capacity.

**Fig 7:**
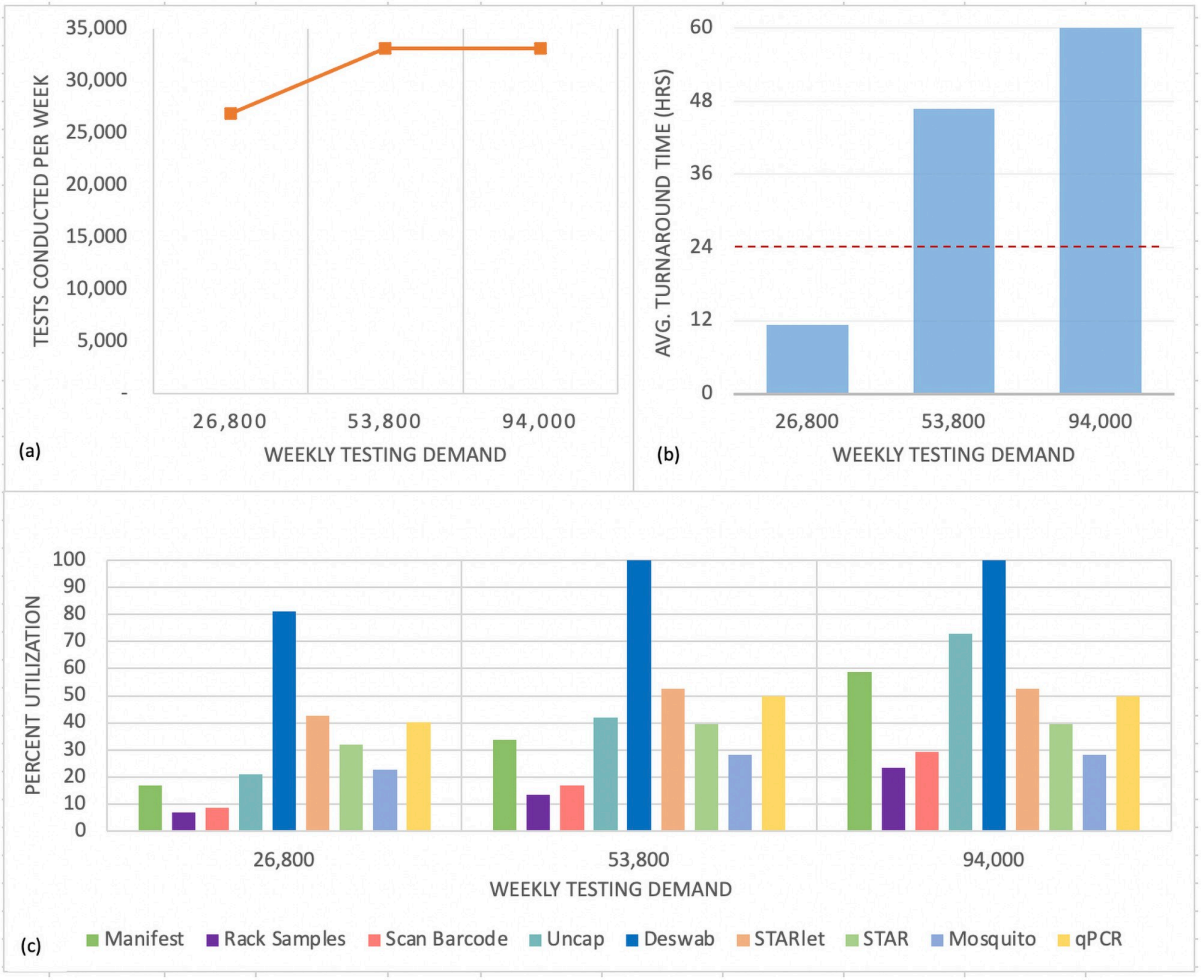
KPIs for the baseline scenario that represents current UMPA/MG operations. (a) Tests conducted per week. (b) Average turnaround time from collection to result. (c) Percent utilization of resources at each process step (e.g., technicians or robots).

First, the baseline scenario was simulated with multiple levels of demand, to determine the impact of different testing demand levels on the KPIs and to identify the lab’s overall capacity. Demand levels were set to correspond to a typical week, double the demand of a typical week, and more than triple the demand of a typical week (to significantly stress the system). The results are shown in Fig 7. Fig 7a shows that the maximum capacity is about 33,000 samples per week. Fig 7b shows that when the laboratory is operating at or below its capacity, the result turnaround time is well under 24 hours (its target). However, when demand for testing exceeds the laboratory’s capacity, the result turnaround time grows beyond 24 hours because samples are backed up waiting for processing.

Fig 7c shows which step is the bottleneck that constrains the overall capacity of the laboratory. The percent utilization is the percentage of time that any given process step is “busy”: for example, the percentage of time that the STARlet robot is being used to process samples. When a process step is busy nearly 100% of the time, it is acting as a bottleneck, because samples are likely queuing to wait for their turn in that step. Fig 7c shows that the deswabbing step is utilized at a much higher rate than the other steps, and reaches 100% utilization at the medium demand scenario. Therefore, it is acting as the bottleneck.

**Fig 8:**
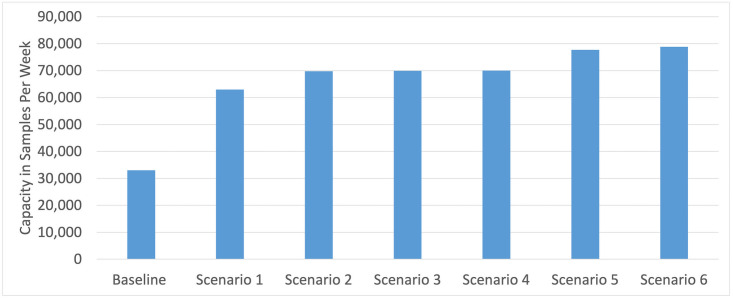
Changes in capacity resulting from various process modifications.

With a bottleneck identified, the next step is to consider alleviating this bottleneck to expand the overall capacity of the laboratory. After one bottleneck is alleviated, others will arise that limit the laboratory’s capacity to a different (higher) number of tests per week. We ran a series of what-if scenarios to find and eliminate successive bottlenecks, following the same approach as the analysis just described. The strategies for eliminating each bottleneck were suggested by laboratory stakeholders. The analyses and results are summarized in Table 2. Fig 8 shows the changes in capacity as successive bottlenecks are eliminated.

**Table 2:** Capacity and bottlenecks with various process modifications.

No.	Scenario	Assumptions	Capacity (tests per week)	Bottleneck(s)
0	Baseline	See Table 1	33,000	Deswabbing
1	Only 30% of samples need deswabbing	For 70% of the samples, deswabbing is not needed because swabs are discarded when samples are collected	63,000	Sample transfer to deep-well plates (STARlet)
2	Only 30% of samples need deswabbing ***and*** repurpose one STAR to transfer samples to deep-well plates	Same as Scenario 1, *and* one STAR instrument is repurposed to transfer samples to deep-well plates (rather than RNA extraction)	69,800	Sample transfer to deep-well plates (STARlet), RNA extraction (STAR), qPCR
3	Only 30% of samples need deswabbing ***and*** repurpose one STAR to transfer samples to deep-well plates ***and*** multiplex assay	Same as Scenario 2, *and* multiplex the assay to use 2 wells instead of 3 wells, which doubles the throughput at qPCR	69,900	Sample transfer to deep-well plates (STARlet), RNA extraction (STAR)
4	Only 30% of samples need deswabbing ***and*** repurpose one STAR to transfer samples to deep-well plates ***and*** 10-hour shifts for upstream process	Same as Scenario 2, *and* the shift length for all “upstream” steps -- those from the start of the lab process up to and including sample transfer to deep-well plates (STARlet) -- is extended to 10 hours.	70,000	RNA extraction (STAR), qPCR
5	Only 30% of samples need deswabbing ***and*** 10-hour shifts for upstream process	Same as Scenario 1, *and* the shift length for all “upstream” steps -- those from the start of the lab process up to and including sample transfer to deep-well plates (STARlet) -- is extended to 10 hours.	77,700	Sample transfer to deep-well plates (STARlet), qPCR
6	Only 30% of samples need deswabbing ***and*** 10-hour shifts for upstream process ***and*** multiplex assay	Same as Scenario 5, *and* multiplex the assay to use 2 wells instead of 3 wells, which doubles the throughput at qPCR	78,800	Sample transfer to deep-well plates (STARlet)

Table 2 and Fig 8 show that deswabbing is the most critical bottleneck: reducing the need for deswabbing by 70% (or, equivalently, increasing deswabbing capacity) can almost double the capacity of the entire laboratory, from 33,000 to 63,000 tests per week (Scenario 1). With different amounts of reduction in deswabbing requirements or increases in deswabbing capacity (not shown), the lab’s overall capacity changes fairly linearly until it reaches the next bottleneck.

Once the need for deswabbing is reduced to around 30% of samples, a new bottleneck arises: the capacity of the STARlet robots to transfer samples to deep-well plates. A further slight increase in capacity can be achieved by repurposing a STAR robot to perform this function (Scenario 2). Further significant increases in capacity require investing in additional personnel time and/or robots to alleviate simultaneous bottlenecks at three steps: sample transfer to deep-well plates (STARlet), RNA extraction (STAR), and qPCR. For example, a further moderate increase can be achieved by increasing shift lengths to 10 hours for just the upstream process steps (up to and including the STARlet) and multiplexing the assay to double the PCR capacity. Further increases in capacity would require more significant investments in additional robots and/or personnel time, or a new “evening shift” of workers so that existing equipment could be put to use for more of the hours in each 24-hour period.

### Analysis 2: Impact of demand variability

The second analysis examines the impact of variability in daily demand for testing on the performance of the laboratory. Recall that the number of samples arriving at the laboratory for testing each day varied widely (Fig 4). Analysis 1, above, assumed approximately constant demand for testing each day; this analysis shows how variability impacts the laboratory’s performance.

For clarity, we analyze a simple variability scenario, in which a single large “spike” in demand is seen on day 3 of the analyzed week, while the demand on other days remains constant (at the daily level seen in a typical week with a demand of 26,800). This was compared to a scenario with the same total weekly demand (including the spike) spread evenly across all the days.

**Fig 9:**
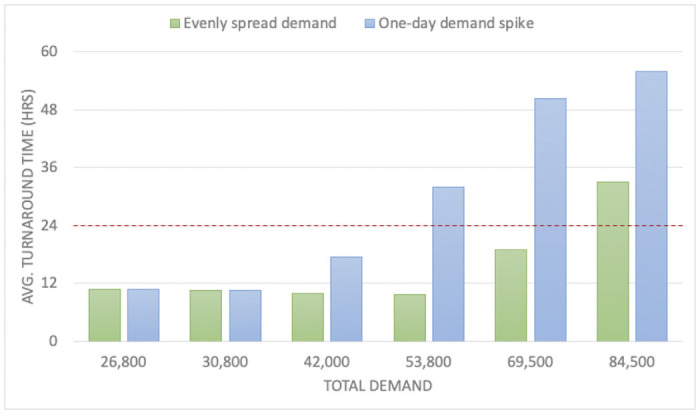
Average turnaround time for demand scenarios with one-day spikes compared to evenly spread demand, for Scenario 1.

The results show that this variability in demand has a major impact on the turnaround time for returning results. Fig 9 shows the turnaround times for Scenario 1 (described in Table 2), where only 30% of samples need deswabbing. When the demand is highly variable, i.e., when it includes a one-day spike, the turnaround times exceed the 24-hour target at much lower weekly demand levels than when the demand is evenly spread across the week. Therefore, although Analysis 1 found that Scenario 1’s weekly capacity is 63,000, large variability in demand *reduces* its effective capacity if 24-hour turnaround times are required -- in this case, to less than 53,800. Thus, demand variability increases turnaround times when the total demand is near the total capacity of the lab. Once samples are backed up waiting for a process step to be available, it can be hard to “catch up” as new samples are continually arriving. Therefore, when large variability in demand is expected, more capacity is needed if reliable 24-hour turnaround times are desired. The average utilization rate of each process step should be much lower than 90% -- in other words, some steps will not be busy some of the time -- so that there is sufficient capacity to handle high-demand days while maintaining 24-hour turnaround times.

### Analysis 3: Minimum resources to meet a target capacity

The third analysis determines the minimum set of resources (people and instruments) required for a laboratory to meet a target weekly testing capacity. The results provide a guide for scaling up capacity for any laboratory following a sufficiently similar process.

We define a “resource unit” as the smallest possible unit of capacity that could be added to the process. For example, a “resource unit” is a single robot/instrument or a single full-time lab technician if the process step is manual. (Technician hours could be reduced below full time, or smaller sizes of robots/instruments could be purchased, but these options were not considered in this analysis.)

The same three levels of weekly demand are considered as in Analysis 1, and these are treated as targets. For each target, a series of what-if scenarios were evaluated in the model. The first scenario contained just one resource unit for each step in the process. The succeeding scenarios added single resource units for any steps that were utilized more than 90% of the time (because they were acting as a bottleneck and constraining system capacity). The process was repeated until all process steps were utilized less than 90% of the time. The resulting number of resource units represents the minimum resources required to meet the target weekly demand. The same analysis was also performed with a utilization threshold of 70%, which provides better robustness to variability in demand.

The results are shown in Table 3. These results can guide the laboratory in making investments in instruments and hiring to meet a particular target capacity. For example, the lab currently has sufficient instruments to meet a target capacity of 53,800, but not enough deswabbing technicians. To meet this target capacity, they would need to hire (or repurpose) two additional technicians for deswabbing. These results are also useful beyond the laboratory we studied, as a benchmark for resource investment, to the extent that the process steps and resource units are similar across laboratories. This is likely to be the case, since the same fundamental steps are always carried out, but there are some important potential variations. First, there are multiple options for robotic liquid handling robots, which could replace the Hamilton STAR and STARlet, and they might have different capacities. Second, different assays utilize different numbers of wells on the plates, and different laboratories may use different plate sizes. It is relatively simple to modify the model to account for these differences, but there are too many possibilities to analyze in this paper.

**Table 3:** Minimum resources required to meet target levels of demand (8-hour shifts).

Target Capacity	Utilization Threshold	Capacity of Process Step (Resource Units)						
		Man.+ Rack+ Scan (Techs)	Uncap (Techs)	Deswab (Techs)	STARlet* (Instr.)	STAR* (Instr.)	Mosq.* (Instr.)	qPCR* (Instr.)
26,800	90%	1	1	2	2	3	1	8
26,800	70%	1	1	2	2	3	1	10
53,800	90%	2	1	4	3	5	2	15
53,800	70%	2	2	5	4	6	2	18
94,000	90%	3	2	7	5	8	3	26
94,000	70%	3	2	8	7	10	4	32

* Instruments also require technicians to run the instruments, but a single technician can run several instruments at once. These technicians are not accounted for in this analysis.

### Sensitivity to key assumptions

The model is particularly sensitive to the assumed time it takes to deswab a sample tube, because this is a major bottleneck. This assumption is also particularly uncertain. Experts noted that the technicians had gotten much faster at deswabbing over time and that the time also depended on the characteristics of the swab and sample tube, which varied because multiple different products were used. In applying these results, it is important to consider the potential differences in deswabbing times and the level of experience of the operator.

The capacity of the laboratory also depends strongly on the assumed shift length per day. The results can be scaled for different shift length assumptions, since the capacity will scale with the shift length.

## Discussion and conclusions

Testing for COVID-19 in support of large-scale public health needs, such as surveillance and mitigation, represents a quintessential complex adaptive system, as illustrated in Fig 1. Recent scholarly work has emphasized the value of systems thinking for responding to the COVID-19 pandemic [e.g., 16–19]. The current testing response, with capacity shortages and long turnaround times, suggests gaps in operational planning and decision making that obstructed rapid and effective service delivery in a complex and somewhat weakened hybrid public/private health system.

To address this gap, we explored one aspect of the testing system by evaluating the impact of the dynamics of the COVID-19 pandemic on the needs and capacity for testing at a large, retrofitted academic lab—one of many that were required to quickly pivot from a research to a public service laboratory. Using simulation models of the testing process, we were able to make both specific process adaptation recommendations and elucidate general insights into COVID-19 testing dynamics by evaluating different what-if scenarios. When calibrated to the characteristics of the operational protocols from a particular laboratory, what-if scenarios informed performance gains or losses (in capacity and turnaround time) when making various changes to (a) the *process itself* and/or (b) the *resources* assigned to the process, or (c) when dealing with changes in the *environment*.

For example, when Analysis 1 showed that a near-doubling of capacity could be achieved by alleviating a key bottleneck at deswabbing, the lab investigated different strategies to address this, including hiring additional personnel or asking collection sites to discard swabs rather than leaving them in sample tubes. The latter was chosen because it was cheaper, after the laboratory found a way to avoid biohazard disposal requirements at testing sites by using a collection buffer that inhibits the virus on contact. Similarly, instruments with lower utilization rates were repurposed to perform a different function after our results showed potential gains in capacity, obviating the need to purchase new capital equipment to meet a temporary increase in utilization. A broader principle illustrated by these analyses is that simple process changes can make a profound performance difference without significant financial investment. On the other hand, other process changes were unexpectedly *un*-impactful. Multiplexing the assay to double the throughput at the qPCR step showed very little potential to improve the overall laboratory capacity because this step was not a key bottleneck at this laboratory, which already had plenty of PCR machines (though multiplexing could still improve cost and supply requirements). Because the model predicted limited impact, the laboratory did not need to make the investment to design and deploy a multiplexed assay.

A second principle illustrated by our findings is that high variability in demand for testing, which is inevitable in a rapidly evolving pandemic situation, has a large impact on performance. When trying to achieve stable, short turnaround times, planning for this variability and building in buffer capacity is crucial to system resilience.

These results and this approach are not limited to just one specific laboratory. The steps in the RT-PCR testing process are similar across most laboratories, and while there may be minor differences in the equipment and batch sizes, broad generalizations—or “rules of thumb”—can be distilled from the simulation output. For example, for labs performing similar processes (e.g., automated liquid handling, RNA extraction, and RT-PCR), the analysis of minimum resources required to meet a target capacity (Analysis 3) may be used as a benchmark for planning. Where necessary, it is also straightforward to adapt the model directly to other laboratory operational processes. Relatively little data would be needed, such as: instrument capacities, number of instruments, number of staff, time to perform each step, batching and pooling strategies, shift lengths, and positivity and inconclusivity rates (see S1 File). These data should be readily available or easily measurable at most laboratories. Even in circumstances where developing or adapting a model to a different operational protocol is not feasible, our work shows the relative ease and value of using our analytical approach to evaluate the testing process and resources. Specifically, we have clarified the key performance indicators and the fundamental process steps. The KPIs could be routinely collected and monitored in testing laboratories to identify bottlenecks whose alleviation could improve capacity or speed results return.

In general, our pilot experience demonstrates the utility of process simulation models as decision support tools in this context. They can highlight multiple ways to achieve performance improvements, and simulate the impact of using one versus another using readily available data, to surface and quantify the trade-offs associated with each option (in performance, cost, and complexity). These results may then be used to guide decision-making. By using the simulation models as decision support tools, the impact of a rapidly changing context (e.g., variability in testing demand and resource availability) can be rapidly investigated, enabling decision-making even in the face of uncertainty. The models also enabled decision-makers to move past strong opinions and assumptions to explore their options using data-informed model outputs. Critically, simulation outputs allow decision-makers to virtually ‘test’ different adaptations to the system before a significant (and possibly hard to reverse) investment or disruption to real operational processes is implemented. Our pilot work provides proof of principle that stakeholders gained insight and value from the simulation models and the meetings and workshops in which the results were discussed. Our experience suggests that similar participatory process modeling efforts will yield high value to current and future pandemic response and preparedness, as well as to increased efficiencies in screening and diagnostic testing in public health and healthcare settings in the United States and abroad.

Moving forward, we recognize many opportunities to refine and broaden this analysis of operational processes for COVID-19 RT-PCR testing. One priority is to analyze additional process changes in the laboratory, such as pooled testing [11, 12] and prioritization of specific groups of samples [8–10]. A second priority is to explore the impact of other environmental conditions, such as wider variation in demand and processing times, as might be seen during scale-up or in low- and middle-income countries. A third priority is to further characterize the impact of demand variability, and to develop a generalizable “rule of thumb” for the appropriate target utilization rate for process steps, based on the variability in testing demand. A fourth priority is to refine and validate the generalizability of the laboratory process model to enable the development of an online ‘dashboard’ to help other laboratories plan for deployment of public health services as needed. Finally, for this pilot study, we focused almost exclusively on the processes within the testing laboratory. Future work to extend the boundaries to integrate the processes from other elements of the testing (Fig 1), including sample collection and response to positive tests (e.g., quarantine), can provide insight into how operational decisions in each sector impact the others and ultimately impact the overall effectiveness of COVID-19 testing in mitigating community spread of infection. This would enable a more holistic view of tradeoffs between testing capacity investment and other mitigation measures, and show what the target capacity *should* be and *can* be under different resource scenarios to meet the desired goals of mitigation and containment.

Despite ready access to state-of-the-art molecular technologies, COVID-19 RT-PCR testing capacity has fallen far short of the need in the United States to allow for a safe re-opening of society during the COVID-19 pandemic. In preparation for a potential future pandemic, it is crucial to learn from these shortcomings and to set up policies, operational plans, and resources for rapid scale-up of testing capacity. This study has demonstrated a valuable but under-utilized role for operations research in this effort, and provided an approach that could be used to study other laboratories operating under different contexts. Applying the approach broadly could facilitate a retrospective evaluation of testing processes and their performance. Such a “forensic” analysis, combined with forward-looking scenario analyses, would provide valuable insights for preparedness planning and standard operating procedures to facilitate a future rapid deployment of a “public health testing reserve” in the event of future pandemic threats.

## Supporting information

S1 File Modeling assumptions. Details the assumptions used in the model.
(PDF)
