# Supplementary material for: Supporting scale-up of COVID-19 RT-PCR testing processes with discrete event simulation
Source: PLoS One. 2021 Jul 29;16(7):e0255214. doi: 10.1371/journal.pone.0255214 (PMC8321135; doi:10.1371/journal.pone.0255214)
Supplement: S1 File — Details the assumptions used in the model. (PDF) [file pone.0255214.s001.pdf]

Modeling Assumptions

The following tables detail the assumptions used in the model.

Table S1: Assumptions for processing times, arrival rates, batches and shifts

Parameter	Assumption	Distribution	Units for Distribution
**School/workplace patient arrival rate**	Poisson process ~9,240 arrivals/week	Poisson process ~9,240	arrivals/week
**Community testing patient arrival rate**	Poisson process ~9,240 arrivals/week	Poisson process ~9,240	arrivals/week
**Nursing homes sample arrival rate**	Poisson process ~8,400 arrivals/week	Poisson process ~8,400	arrivals/week
**Match/Create EPIC record processing time**	4 minutes/patient	triangular(3,4,5)	minutes/sample
**Match Requisition (school/workplace) processing time**	30 secs/patient	triangular(25,30,35)	seconds/sample
**Sample Collection (school/workplace) processing time**	30 secs/sample	triangular(25,30,35)	seconds/sample
**Sample Collection (community) processing time**	30 secs/sample	triangular(25,30,35)	seconds/sample
**Sample Accessioning (rapid) (community) processing time**	30 secs/sample	triangular(25,30,35)	seconds/sample
**Sample Accessioning (nursing homes) processing time**	3-5 mins/sample	triangular(3,5,6)	minutes/sample
**Manifest building processing time**	5 mins per 100 samples	triangular(3,3.5,5)	seconds/sample
**Rack Samples processing time**	1-2 secs /sample	triangular(0,1,2)	seconds/sample
**Scan Barcode processing time**	30 secs/rack of 24 samples	triangular(24,30,36)	seconds/rack
**Uncapping processing time**	3 secs/sample	triangular(67.2,72,84)	seconds/rack
**Deswabbing processing time**	3-30 secs/sample	triangular(1.2,3,10)	minutes/rack
**STARlet processing time**	15 mins/plate of 96 samples	triangular(14.5,15,15.5)	minutes/plate
**STAR processing time**	1.5 hours/ 4x plates of 96 samples	triangular(1.45,1.5,1.55)	hours/plate
**Mosquito processing time**	8 mins/batch	triangular(7.8,8,8.5)	minutes/plate
**qPCR processing time**	1.25 hours/batch	triangular(1.24,1.25,1.3)	hours/batch

**Sample Retrieval processing time**	1.25 minutes / sample	triangular(1.1,1.25,1.3)	minutes/sample
**Export Data processing time**	4 mins/ batch	triangular(3.5,4,4.5)	minutes/batch
**Manual Verification processing time**	15 secs/ sample	triangular(15,16,17)	seconds/sample
**Rack Sample batch size**	24 samples per batch	24	samples
**STARlet batch size**	96 samples per batch	96	samples
**STAR batch size**	384 samples per batch	384	samples
**qPCR batch size**	96 samples per batch	96	samples
**Shipping from Colleges batch size**	250 samples per batch	triangular(100,250,600)	samples
**Shipping form community testing batch size**	250 samples per batch	triangular(100,250,600)	samples
**Positivity rate**	1.20%	1.2	percent
**Inconclusive test rate**	2.30%	2.3	percent
**Lab shift length**	8 hours	8	hours
**Accessioning shift length**	24 hours	24	hours

Table S2: Assumptions for number of resources

Resource Capacities	Assumption
**Sample Accessioning (rapid) (community)**	33 technicians
**Sample Accessioning (nursing homes)**	33 technicians
**Manifest Building**	2 supervisors
**Rack Samples**	2 technicians
**Scan Barcode**	2 technicians
**Uncapping**	2 technicians
**Deswabbing**	2 technicians
**STARlet**	3 instruments
**STAR**	6 instruments
**Mosquito**	3 instruments
**qPCR**	16 instruments
